# NIR Absorbing AzaBODIPY Dyes for pH Sensing

**DOI:** 10.3390/molecules25163689

**Published:** 2020-08-13

**Authors:** Gugu Kubheka, John Mack, Tebello Nyokong, Zhen Shen

**Affiliations:** 1 Institute for Nanotechnology Innovation, Department of Chemistry, Rhodes University, Grahamstown 6140, South Africa; gugu_kubheka@yahoo.com (G.K.); t.nyokong@ru.ac.za (T.N.); 2 State Key Laboratory of Coordination Chemistry, School of Chemistry and Chemical Engineering, Nanjing University, Nanjing 210046, China

**Keywords:** aza-BODIPY dye, pH sensing, intramolecular charge transfer, photophysical properties, pK_a_ values

## Abstract

Two near-infrared (NIR) absorbing di(thien-2-nyl)-di(dimethylanilino)azaBODIPY dyes **2a** and **2b** were synthesized and characterized that differ depending on whether the dimethylaniline substituents are introduced at the 3,5- or 1,7-positions of the azaBODIPY core. The main spectral bands lie at 824 and 790 nm, respectively, in CH_2_Cl_2_. The effect of substituent position on the photophysical and pH sensing properties was analyzed through a comparison of the optical properties with the results of time-dependent density functional theory (TD-DFT) calculations. Protonation of the dimethylamino nitrogen atoms eliminates the intramolecular charge transfer properties of these compounds, and this results in a marked blue-shift of the main absorption bands to 696 and 730 nm, respectively, in CH_2_Cl_2_, and a fluorescence “turn-on” effect in the NIR region. The pH dependence studies reveal that the pK_a_ values of the non-protonated **2a** and **2b** molecules are ca. 6.9 (±0.05) and 7.3 (±0.05), respectively, while that of the monoprotonated species for both dyes is ca. 1.4 (±0.05) making them potentially suitable for use as colorimetric pH indicators under highly acidic conditions.

## 1. Introduction

Near-infrared (NIR) region absorbing dyes are essential for various applications in fields as diverse as materials science and medicine [1]. NIR dyes are advantageous in a biomedical context due to reduced background absorption, fluorescence, and light scattering, resulting in improved sensitivity [2]. Boron azadipyrromethene (azaBODIPY) dyes are of particular interest, since the incorporation of an aza-nitrogen atom into the boron dipyrromethene (BODIPY) chromophore is one of the main strategies used to red shift the main spectral band into the NIR region [3]. Among the various possible NIR chromophores, there has been an increasing focus on azaBODIPY dyes in recent years due to their favorable photophysical properties, such as high photostability, and their structural versatility and ease of modification at all positions on the azaBODIPY core [4,5]. Different strategies have been used to develop dyes with highly red-shifted spectral bands, such as attaching electron-donating and withdrawing group at the 3,5- and 1,7-positions, respectively; replacing the phenyl groups with smaller five-membered heterocyclic rings [5,6,7,8]; and the addition of fused rings to the pyrrole rings of the azaBODIDY core [9]. When electron-donating aryl groups, such as dimethylaniline, are introduced at the 3,5-positions there is a large red-shift of the main spectral band, since there is a marked decrease in the HOMO–LUMO gap [10]. The red-shifts that are observed when a five-membered ring system is incorporated into the structure have been ascribed to increased delocalization that arises from enhanced co-planarity with the azaBODIPY core and reduced torsion angles relative to structures that contain phenyl rings [5]. Substitution with thiophene rings also provides scope for more effectively conjugated donor-acceptor compounds [11].

The inclusion of dimethylaniline substituents into the structures of azaBODIPY dyes can generate pronounced photophysical changes as a result of substrate recognition [12,13], due to changes in the intramolecular charge transfer (ICT) properties of the dyes. Therefore, this ICT switching mechanism can potentially be used to form pH indicators [12] or in the detection of small metal ions such as Ca^2+^, Cu^2+^, and Mg^2+^ when a more elaborate amine receptor is used [14]. ICT systems are advantageous for sensor applications, since the use of highly fluorescent dyes results in inner-filter effects, due to the bias introduced by the pH-dependent emission [14]. A limited number of pH-sensitive amino moiety functionalized azaBODIPY dyes have been previously reported as indicator dyes, and different spectroscopic methods, such as absorption and fluorescence, have been used to measure the pH of the probes [1,12,14]. For example, McDonnell et al. reported the sensing properties of 1,7-diphenyl-3,5-di-(dimethylanilino)azaBODIPY (**1**), which is structurally similar to the dyes reported herein [12].

**Figure 1:**
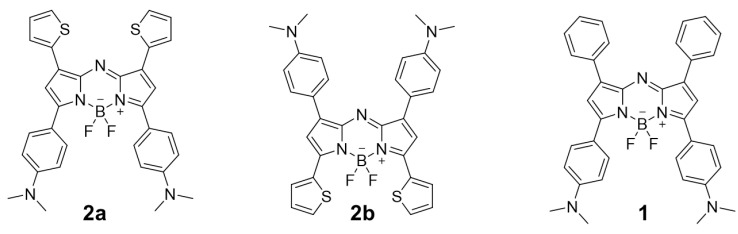
A comparison of the molecular structures of **2a** and **2b** with that of **1**, which was reported previously by McDonnell et al. [12].

In this study, we report the synthesis of dimethylaniline and thien-2-yl substituted aza-BODIPY dyes **2a** and **2b** as NIR and pH-responsive chromophores by varying the position of the substituents between 1,7- and 3,5-positions (Figure 1). The aim of the study is to investigate the effect of the substituent and position on the photophysical properties and pK_a_ values, and to form azaBODIPY dyes with spectral bands red shifted beyond 750 nm. To the best of our knowledge, there have been very few reports on non-protonated azaBODIPY dyes that exhibit no fluorescence emission and hence provide scope for use as “turn-on” fluorescence pH sensing indicators in the NIR region. The goal was to use the ICT properties associated with the dimethylaniline groups to achieve this.

## 2. Results

### 2.1. Synthesis

**Scheme 1:**
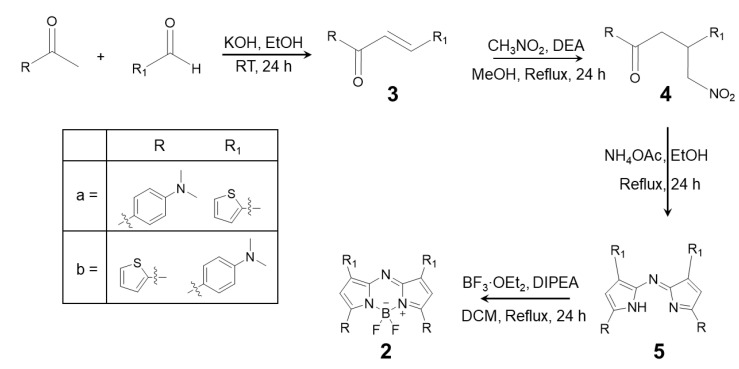
Synthesis of azaBODIPYs **2a** and **2b**.

AzaBODIPYs **2a** and **2b** were synthesized according to literature procedures (Scheme 1) [5,15,16]. After initially forming chalcone **3** from the appropriate aryl aldehyde and acetophenone and subsequently converting it to nitro-derivative **4**, azadipyrromethene **5** was obtained by heating **4** under reflux with ammonium acetate (NH_4_OAc) in *n*-butanol (*n*-BuOH) for 24 h. After separation on column chromatography, the BF_2_ moiety was introduced to **5** using boron trifluoroetherate (BF_3_·OEt_2_) and diisopropylethylamine (DIPEA) in CH_2_Cl_2_ under reflux for 6 h. The products were isolated as bluish-purple and blue for **2a** and **2b**, respectively, in moderately high yields. The target compounds were characterized by ^1^H NMR and FTIR spectroscopy and mass spectrometry. The ^1^H NMR spectrum of **2a** (Figures S1 and S2, Supporting Information) contains peaks at 7.24 (double doublet), 7.69 (double doublet) and 8.06 (double doublet) ppm that can be assigned on the basis of integration to the thien-2-yl groups, while the corresponding peaks for **2b** lie at 7.23 (double doublet), 7.54 (doublet) and 8.32 (doublet) ppm (1-, 2- and 3-H in Figures S1 and S2, Supporting Information). Singlets at 7.37 and 6.99 ppm, respectively, can be attributed to the 2,6-positions of the aza-BODIPY core (4-H in Figures S1 and S2, Supporting Information). Two doublets at 6.86 and 8.22 ppm were detected in the spectrum of **2a** with coupling constants of 9.6 Hz indicating the presence of two adjacent protons (5- and 6-H in Figure S1, Supporting Information) that can be assigned to the dimethylaniline moiety, while the corresponding peaks for **2b** peaks lie at 6.77 and 8.07 ppm (5- and 6-H in Figure S2, Supporting Information). The methyl protons (7- and 8-H in Figures S1 and S2, Supporting Information) lie at 3.15 ppm for **2a** and 3.09 ppm for **2b**. The chemical shifts for **2a** lie upfield of those observed for **2b**. This provides an indication of more efficient ICT from the donor to the acceptor group. Molecular ion peaks are observed at *m*/*z* = 594.96 for **2a** and 595.00 for **2b** (Figures S3 and S4, Supporting Information) in the MS data in close agreement with the calculated value of *m*/*z* = 595.19.

### 2.2. Photophysical Properties

**Figure 2:**
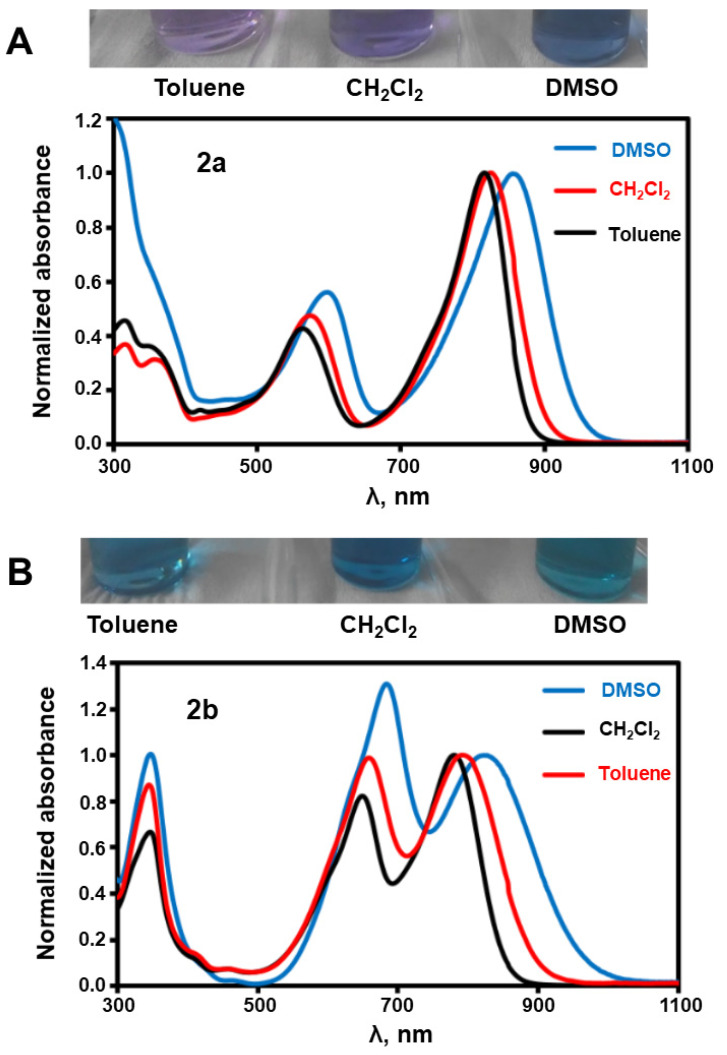
Solvatochromism effects on the optical properties of **2a** and **2b**. Normalized UV-visible absorption spectra of (**A**) **2a** and (**B**) **2b** in dimethylsulfoxide (DMSO), CH_2_Cl_2_ and toluene.

The photophysical properties of **2a** and **2b** were investigated by UV-visible absorption and fluorescence emission spectroscopy in solvents of differing polarity. The absorption spectra of **2a** and **2b** consist of two prominent bands that lie in the 570–600 and 810–860 nm regions for **2a** and 660–690 and 770–850 nm regions for **2b** (Figure 2). In comparison to the spectrum of **1** in toluene [12], the absorption band maximum of the main spectral band for **2a** is red shifted by 18 nm (Table 1).

Both **2a** and **2b** exhibit marked solvatochromism (Figure 2 and Table 1). The solvatochromism is stronger for **2b** where the dimethylaniline groups are substituted at the 1,7-positions, since the ICT character of the S_1_ state is expected to be particularly strong due to the effect of the electron-donating effect of these substituents on the HOMO which is expected to have large MO coefficients at the 3,5-positions [17]. Both dyes exhibit linear Beer-Lambert plots in all solvents studied, which points to an absence of aggregation. In contrast with **1** [12], when the fluorescence properties of **2a** and **2b** were studied in polar, polar aprotic and non-polar solvents, no emission was observed. The fluorescence quenching is related to ICT character in the S_1_ state that is associated with the presence of the electron-donating thien-2-yl and dimethylanilino groups and an electron-deficient azaBODIPY core [18]. The heavy atom effect of the thiophene sulfur atoms is the most likely explanation for differences in the fluorescence properties of **2a** and **2b** relative to those of **1** [12].

### 2.3. Effect of Protonation in CH_2_Cl_2_

**Figure 3:**
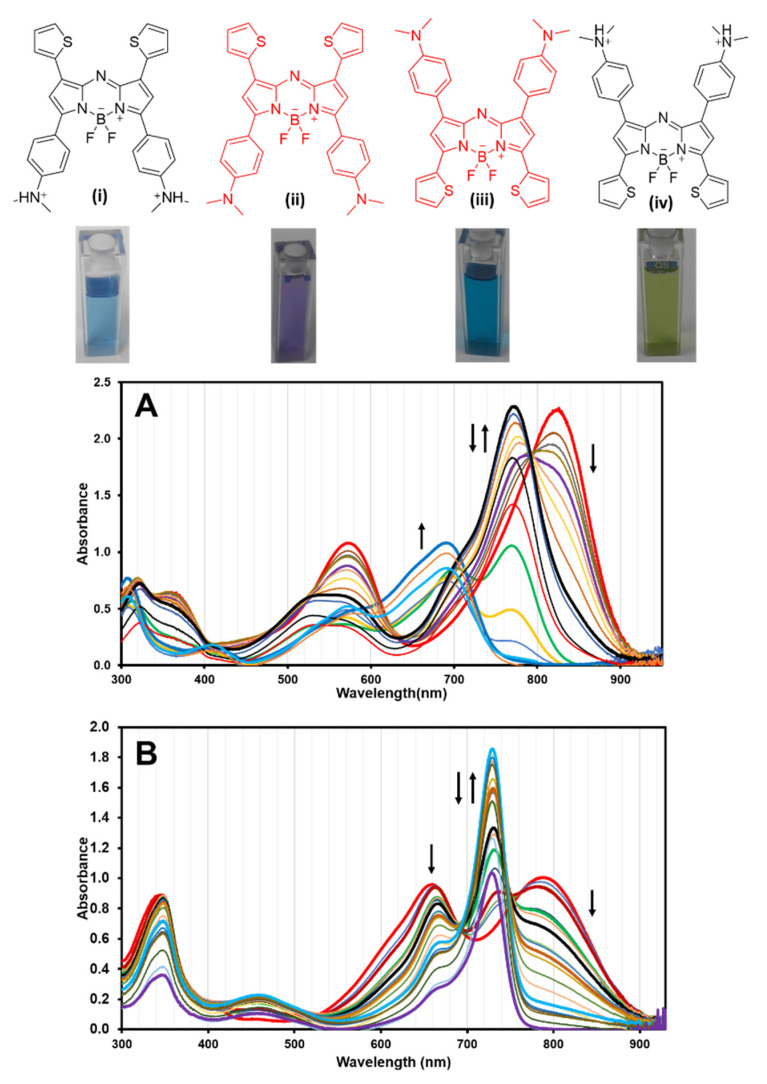
The diprotonated ((**i**) and (**iv**)) and non-protonated ((**ii**) and (**iii**)) structures of **2a** and **2b** and the observed colorimetric changes upon protonation with trifluoroacetic acid TFA (TOP). Effect of protonation (BOTTOM) on the absorption spectra of **2a** (**A**) and **2b** (**B**) in CH_2_Cl_2_.

The effect of adding trifluoroacetic acid (TFA) to solutions of **2a** and **2b** in CH_2_Cl_2_ on the absorption and emission spectra was investigated. Protonation of **2a** and **2b** is accompanied by visible color changes, as shown in (Figure 3). O’Shea et al. have demonstrated that protonation disrupts the dimethylamino lone pair and this eliminates the ICT character of the S_1_ state, and this results in a blue shift of the main spectral band and diminished absorbance [19]. In a similar manner, the addition of TFA to CH_2_Cl_2_ solutions of **2a** and **2b** results in a large blue shift of the main absorption bands (Figure 3). Upon addition of TFA to the CH_2_Cl_2_ solution of **2a** solution, new bands are observed at 772 and 694 nm representing mono- and diprotonated species, respectively. The non-protonated species band at 824 nm gradually diminishes in intensity. The reasonably sharp isosbestic points at 790 and 746 nm provide an indication that points of equilibrium are reached during the addition of TFA (Figure 3). When TFA is added to the CH_2_Cl_2_ solution of **2b**, a new band appears at 731 nm. The spectra of the mono- and diprotonated species are not as well defined in this case.

**Figure 4:**
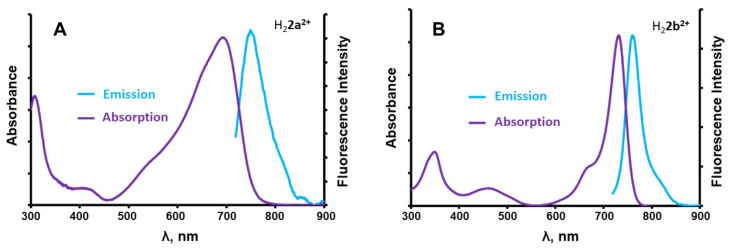
Absorption and emission spectra of H_2_**2a**^2+^ (**A**) and H_2_**2b**^2+^ (**B**) in CH_2_Cl_2_ at λ_ex_ = 600 nm.

The fluorescence spectra for H_2_**2a**^2+^ and H_2_**2b**^2+^ in CH_2_Cl_2_ are shown in Figure 4. The fluorescence quantum yields (Φ_F_) were determined to be 0.04 and 0.14 in CH_2_Cl_2_, respectively. Relatively low Φ_F_ values are expected for these compounds, since the values for NIR-emitting dyes are generally lower than for those emitting in the visible region [20]. The absence of fluorescence for **2a** and **2b** provides scope for a clear “turn-on” response for protonation in the NIR region. The response was found to be reversible when triethylamine (TEA) was added to reverse the pH change.

**Figure 5:**
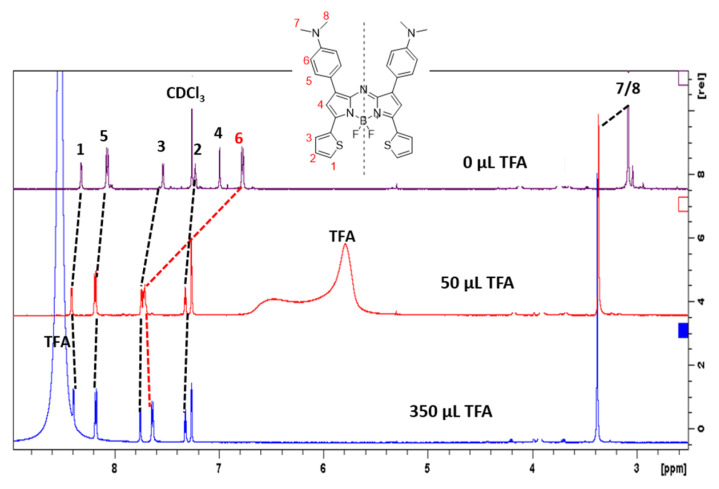
^1^H NMR spectroscopic titration of **2b** with TFA in CDCl_3_.

In addition to the UV-visible absorption and fluorescence titration data, ^1^H NMR spectra were measured in CDCl_3_ to identify the site of protonation on **2b** by using TFA as a titrant. **2b** was sequentially titrated seven times with 50 µL of concentrated TFA to increase the acidity of the solution, and a ^1^H NMR spectrum was obtained at each step. Figure 5 provides a comparison of the ^1^H NMR spectra of **2b** in CDCl_3_ after the initial and final titrations. The initial titration results in a 0.14 M TFA solution and drastically upfield shifted proton signals. It is evident that further titration ultimately resulted in no significant change in the aromatic and the dimethylamino proton signals, except for the deshielding effect induced by protonation. This demonstrates that **2b** only undergoes protonation at the amino nitrogen atoms. The shift of the peak for the dimethylamino moieties from 3.08 to 3.37 ppm demonstrates that protonation occurs at the dimethylamino nitrogens. Initially upon forming the monocation species, rapid exchange of the proton between the two dimethylamino groups results in averaged NMR peaks. Subsequently, both groups are protonated when the dication is formed. The ^1^H NMR spectra for the TFA titration of the dye (Figure S5, Supporting Information) reveal that an unusually broad signal due to TFA shifts across the aromatic region to ca. 8.5 ppm with a simultaneous increase in intensity.

## 3. Discussion

### 3.1. Effect of Protonation in DMSO/Aqueous Solutions

**Figure 6:**
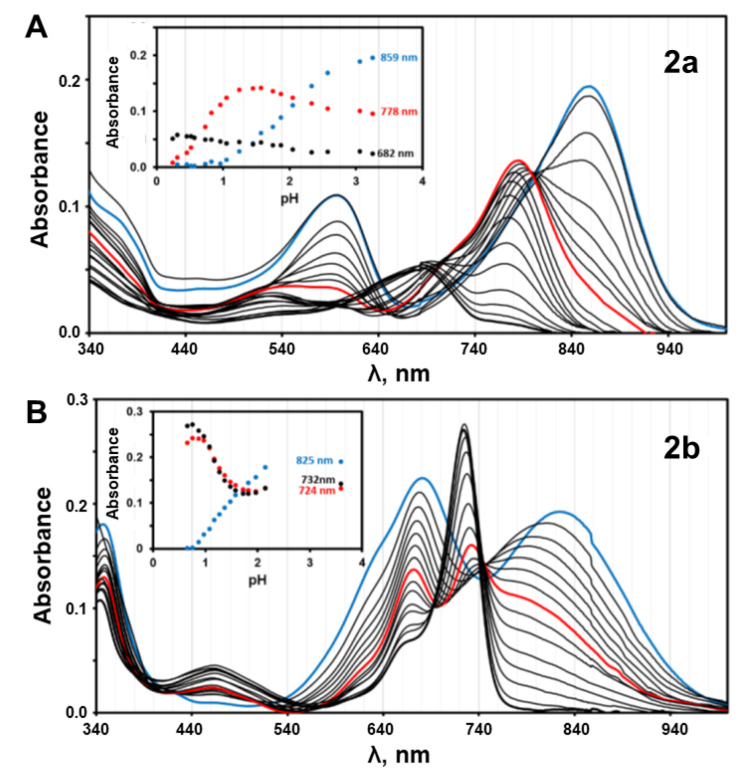
The pH dependence of absorbance for **2a** in 2:1 (*v*/*v*) DMSO/aqueous solution (**A**) and the corresponding calibration curve (inset). The pH dependence of absorbance for **2b** in 2:1 (*v*/*v*) DMSO/aqueous solution (**B**) and the corresponding calibration curve (inset).

Detailed protonation experiments were carried out in 2:1 (*v*/*v*) dimethyl sulfoxide (DMSO)/aqueous solutions, since both dyes are sufficiently soluble in this medium. In principle, the dyes should be suitable for use as acidic range pH indicators in the red/NIR spectral region due to the presence of the dimethylaminostyryl groups at the 1,7- and 3,5-positions. Aliquots of TFA in water were added, and spectral changes were monitored by UV-visible absorption and emission spectroscopy. The main azaBODIPY absorption band was found to undergo a significant red shift to 859 nm in 2:1 (*v*/*v*) DMSO/aqueous solution mixtures relative to what is observed in CH_2_Cl_2_ (Figure 3). The changes in absorption spectra in polar solvents upon protonation were similar to those reported above under TFA titration in CH_2_Cl_2_. There was a marked blue shift of the main absorption band to 778 nm for the monocation species and then to 682 nm for the dication species (Figure 6). Plots of absorbance against pH were used to identify isosbestic points, which are an indication of complete conversion between two distinct forms (Figure 6 insets). Reasonably sharp isosbestic points were observed for **2a** at pH 1.71 (ca. 800 nm) and at pH 1.14 and 0.23 (ca. 700 nm), and for **2b** at pH 1.34 (ca. 745 nm) and pH 1.10 (ca. 700 nm).

**Figure 7:**
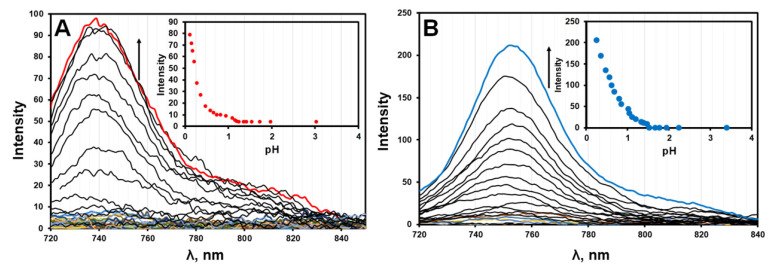
The pH dependence of emission for (**A**) H_2_**2a**^2+^ and (**B**) H_2_**2b**^2+^ in 2:1 (*v*/*v*) DMSO/aqueous solution (1.41 × 10^−6^ M), and the corresponding pH calibration curves at the emission band maxima (insets).

**Figure 8:**
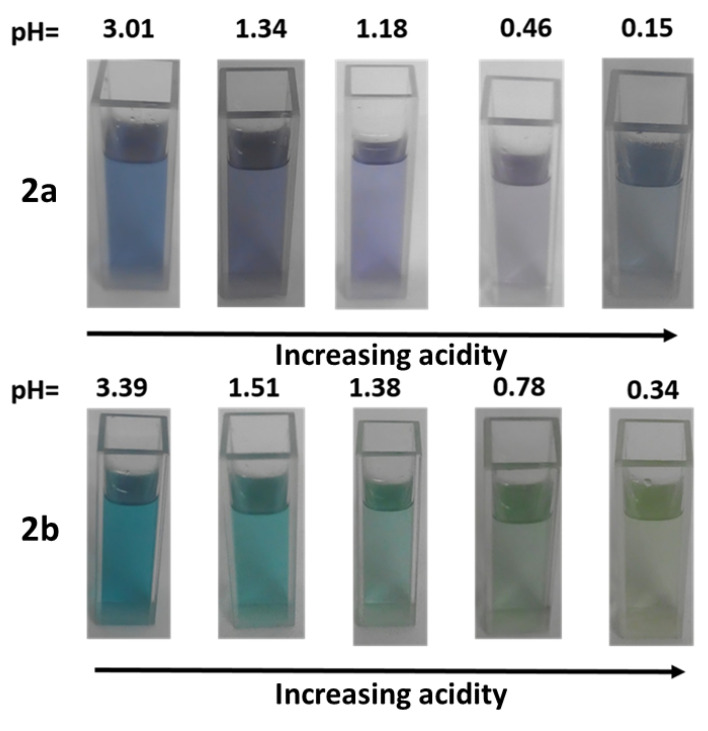
The colorimetric changes for **2a** and **2b** under different pH conditions in 2:1 (*v*/*v*) DMSO/aqueous solutions.

A significant increase was observed in the fluorescence intensity at 742 and 754 nm for **2a** and **2b** when the acidity of the solutions was increased to very low pH values (Figure 7). Visible color changes are observed in this context (Figure 8), making the dyes potentially suitable for use as colorimetric indicators. These changes are consistent with ICT-restricted emission in the presence of dimethylamino groups, which is more pronounced for **2a**.

### 3.2. pK_a_ Determination

**Figure 9:**
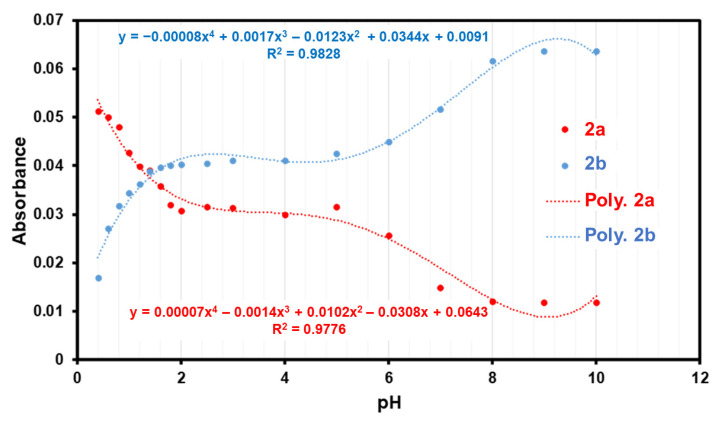
A plot of the observed absorbance values for **2a** and **2b** at 787 and 708 nm, respectively, against pH trend for selected buffer solutions (Table S1, Supporting Information). The best-fit line is to a fourth-order polynomial, the equations are shown, along with the coefficient of determination (R^2^).

The dissociation constant (pK_a_) values were determined to gain insight on their ionization states with respect to pH. Both dyes were prepared in 2:1 (*v*/*v*) DMSO/aqueous buffered solutions (Table S1, Supporting Information), and the absorption measurements were made for the dye:buffer mixtures. Equation (1) was used to obtain approximate pK_a_ values for **2a** and **2b** [21]:

(1) $$pK_{a}=pH+log\frac{d_{M}-d}{d-d_{I}}$$

where pH is the value recorded on the pH meter and d is the absorbance of the molecule in the respective buffers tested. d_M_ and d_I_ are the absorbance of the non-protonated and protonated species, respectively. To verify the pK_a_ results obtained, the inflection method reported was also used [22]. The inflection point is determined from a plot of absorbance against pH, and a best-fit line is obtained by fitting the experimental data with a fourth-order polynomial (Figure 9). The results from both methods were found to be in close agreement. The spectroscopic behavior of both **2a** and **2b** reveals the presence of two pK_a_ values. The pK_a_ values of the non-protonated molecules are 6.9 (±0.05) and 7.3 (±0.05) for **2a** and **2b**, respectively. The monoprotonated species for both dyes has a pK_a_ value of 1.4 (±0.05), which is an increase of 0.1 compared to the apparent pK_a_ value reported by McDonnell et al. [12] for the monoprotonated form of **1** [12]. The relative positions of the thien-2-yl and dimethylaniline groups was therefore found to have a negligible effect on the pK_a_ value in this context.

### 3.3. Molecular Modelling

**Figure 10:**
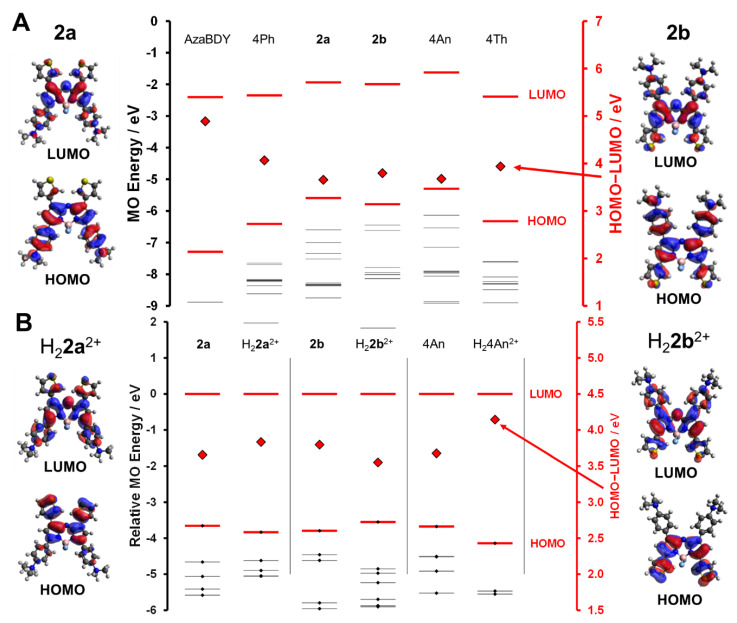
The HOMO and LUMO energies obtained from time-dependent density functional theory (TD-DFT) calculations at the CAM-B3LYP/5-31G(d) level of theory before (**A**) and after (**B**) protonation are highlighted with red lines. When a comparison is made with diprotonated species, the LUMO energy is set to zero. Small black squares are used to denote occupied MOs. Red diamonds are used to highlight the HOMO–LUMO band gap values and are plotted against a secondary axis. The angular nodal patterns of the HOMO and LUMO of the neutral (**A**) and diprotonated (**B**) species of **2a** and **2b** are shown at an isosurface of 0.02 a.u.

Theoretical calculations were carried out for the unsubstituted core dye (AzaBDY), 1,3,5,7-tetraphenyl (4Ph), 1,3,5,7-tetradimethylaniline (4An), and 1,3,5,7-tetrathien-2-yl (4Th) substituted azaBODIPY model complexes, **2a** and **2b** to obtain additional insight about the electronic structures and optical spectroscopy of **2a** and **2b** (Figure 10). The trends predicted in TD-DFT spectra of **2a** and **2b** match those observed experimentally (Table S2, Supporting Information). The incorporation of electron-rich dimethylaniline and thien-2-yl rings makes the azaBODIPY ligand results in a large destabilization of the HOMO energy [23,24]. As can be observed in Figure 10, the substitution of the thien-2-yl rings at the 1,3,5,7-positions of the 4Th model complex results in a slight stabilization of both the HOMO and LUMO relative to the 4Ph model complex, while the presence of electron-donating dimethylaniline rings in the 4An model complex results in a destabilization. In comparison to AzaBDY, the 4Ph, 4An and 4Th model complexes, and **2a** and **2b** are predicted to have significantly smaller HOMO–LUMO energy gaps (Figure 10). The differing arrangements of the substituents at the 3,5- and 1,7-positions of the aza-BODIPY mainly influences the energy of the HOMO. The stronger electron-donating character of the dimethylaniline rings and the presence of large MO coefficients at the 3,5-positions (Figure 10) result in a larger destabilization of the HOMO of **2a** than is the case with **2b**. Protonation of the dimethylamine nitrogen shifts the electron density to the thien-2-yl groups in the HOMO (Figure 10), and this increases the HOMO–LUMO gap in a manner consistent with the observed blue shifts of the main spectral bands (Figure 3, Figure 4 and Figure 6).

## 4. Materials and Methods

### 4.1. Materials

4-Dimethylaminoacetophenone, 4-dimethylaminobenzaldehyde, 2-acetylthiophene, thiophene-2-carboxaldehyde, diethylamine (DEA), potassium hydroxide, nitromethane, potassium carbonate, NH_4_OAc, *n*-BuOH were purchased from commercial suppliers in China. BF_3_·OEt_2_, TFA, TEA, NH_4_OAc, sodium sulfate anhydrous, sodium chloride, potassium hydrogen phthalate, potassium hydrogen phosphate, ethyl actetate, DMSO and DIPEA were purchased from Sigma Aldrich (St. Louis, MO, USA) and used as received. CH_2_Cl_2_, ethanol (EtOH), methanol, potassium chloride and hydrochloric acid were purchased from Minema (Johannesburg, South Africa). Sodium acetate was purchased from Saarchem (Johannesburg, South Africa), and acetic acid was purchased from B&M Scientific (Cape Town, South Africa). All chemicals were analytically pure and were used as received. Acetone-*d*_6_ and CDCl_3_ for ^1^H NMR spectroscopy, and spectroscopic grade solvents for optical spectroscopy were purchased from Sigma-Aldrich and Merck (Darmstadt, Germany), respectively.

### 4.2. Equipment

^1^H NMR spectra were recorded on a Bruker AMX 600 spectrometer (Billerica, MA, USA) at 600 MHz. Mass spectra were determined on a Bruker AutoFLEX III Smartbeam TOF MALDI-TOF mass spectrometer. The instrument was operated in positive ion mode with a 337 nm nitrogen laser and the data were obtained by using α-cyano-4-hydroxycinnamic acid as the matrix. UV-visible absorption spectra were measured on a PerkinElmer Lambda 950 spectrophotometer (Waltham, MA, USA) operated over a wavelength range of 300–1200 nm. Fluorescence spectra were measured on a Varian Eclipse spectrofluorometer (Palo Alto, CA, USA). Fourier transform infrafred (FT-IR) spectra were recorded on an Alpha II (100 FT-IR) spectrometer (Bruker, Billerica, MA, USA) with a universal attenuated total reflectance (ATR) sampling accessory. All the data were obtained from neat particles. The pH values for the buffer solutions were measured using an 86555 Laboratory Benchtop digital pH meter (AZ Instrument Corp., Taichung City, China).

### 4.3. Synthesis

The first step in the preparation of **2a** and **2b** was the preparation of the appropriate chalcones **3** and nitro-derivatives **4** (Scheme 1). Potassium hydroxide (0.16 mol) dissolved in 5 mL water was added at room temperature to a 50 mL solution of the appropriate aryl ketone (11.41 mmol) in ethanol. After stirring for 5 min, an ethanol solution of the appropriate aryl aldehyde (11.41 mmol), and the mixture was stirred for 24 h. The precipitated products were filtered off, washed with aqueous ethanol, and dried. Recrystallization from methanol gave chalcones **3** as light-yellow solids in moderate yields [15]. **3** (100 mmol), nitromethane (500 mmol), and K_2_CO_3_ (0.2 mmol) were dissolved in ethanol (50 mL) and heated to reflux for 12 h. After cooling to rt, the solvent was removed in vacuum and the oily residue obtained was dissolved in ethyl acetate and washed with water three times. The combined organic layers were washed with brine, dried over sodium sulfate, and concentrated to give **4** as yellowish oily residues in nearly quantitative yields, which were used in the next step without further purification [16].

Azadipyrromethenes **5** were used to prepare **2a** and **2b** (Scheme 1). A mixture of **4** (1 mmol) and NH_4_OAc (20 mmol) was refluxed in *n*-BuOH (50 mL) for 24 h. After cooling to rt, the reaction mixture was diluted with water and extracted three times with CH_2_Cl_2_. The combined organic layers were washed with water and brine, dried with sodium sulfate, and concentrated to give the crude intermediate product **5** as a dark blue-black solid, which was then purified by column chromatography prior to use to form the target compounds. DIPEA (0.8 mL, 4.8 mmol) was added to a solution of **5** (1 mmol) in CH_2_Cl_2_ (50 mL), and the mixture was stirred for 1 h. BF_3_·OEt_2_ (0.6 mL, 5.0 mmol) was then added at rt, and the resulting mixture refluxed until the starting material was completely converted (ca. 2 h) to the target product. The progress of the reaction was monitored by thin-layer chromatography (TLC). The cold reaction mixture was diluted with water and extracted twice with CH_2_Cl_2_ (100 mL). The combined organic layers were dried with sodium sulfate and concentrated under vacuum. The crude products were purified by flash column chromatography using CH_2_Cl_2_/hexane (2:1) as eluent. Target compounds **2a** and **2b** were obtained as shiny coppery crystals:

**2a** was obtained in 62% yield; UV-vis (DMSO) λ/nm (log ε) 859 (4.8); IR (FT-IR) ν_max_/cm^−1^: 2952 (=C-H stretch, 858 (-C-H stretch), 1666 (C=N stretch), 1598–1543 (Ar C-C), 1368–1030 (C-N stretch); ^1^H NMR (600 MHz, Acetone-*d*_6_): δ/ppm 3.15 (12H, s, NCH_3_), 6.86 (4H, d, *J* = 9.6 Hz, Ar-H), 7.24 (2H, dd, *J* = 0.9 and 5.1 Hz, Ar-H), 7.37 (2H, s, Ar-H), 7.69 (2H, dd, *J* = 0.9 and 5.1 Hz, Ar-H), 8.06 (2H, dd, *J* = 1.2 and 3.6 Hz, Ar-H), 8.22 (4H, d, *J* = 9.6 Hz, Ar-H); MS (MALDI-TOF): Anal. calc. *m*/*z* 595.19; Found: [M]^+^ 594.96; Anal. calc. for [C_32_H_28_BF_2_N_5_S_2_]: C, 64.54; H, 4.74; N, 11.76; S, 10.77; Found: C, 64.30; H, 4.68; N, 11.80, S, 10.80.

**2b** was obtained in 74% yield; UV-vis (THF) λ/nm (log ε) 786 (4.7); IR (FT-IR) ν_max_/cm^−1^: 2952 (=C-H stretch), 2857 (-C-H stretch), 1667 (C=N stretch), 1598–1543 (Ar C-C), 1368–1030 (C-N stretch); ^1^H NMR (600 MHz, CDCl_3_): δ/ppm 3.09 (12H, s, NCH_3_), 6.77 (4H, d, *J* = 9.0 Hz, Ar-H), 6.99 (2H, s, Ar-H), 7.23 (2H, dd, *J* = 3.6 and 4.8 Hz, Ar-H), 7.54 (2H, d, *J* = 4.2 Hz, Ar-H), 8.07 (4H, d, *J* = 9.0 Hz, Ar-H), 8.32 (2H, d, *J* = 3.6 Hz, Ar-H); MS (MALDI-TOF): Anal. calc. *m*/*z* 595.19; Found: [M]^+^ 595.00; Anal. calc. for [C_32_H_28_BF_2_N_5_S_2_]: C, 64.54; H, 4.74; N, 11.76; S, 10.77; Found: C, 64.50; H, 4.60; N, 11.68, S, 10.82.

### 4.4. Photophysical Parameters

A comparative method was used to determine fluorescence quantum yield (Φ_F_) values:

(2) $$\Phi_{F}=\Phi_{F\left(std\right)}\times \frac{F\;A_{std\;}\eta^{2}}{F_{std\;}A\;\eta^{2}{}_{std}}$$

where F and F_std_ are the areas under the fluorescence curves of **2a** and **2b** and the reference, respectively. A and A_std_ are the absorbances of sample and reference at the excitation wavelength, and η^2^ and η^2^_std_ are the refractive indices of solvents used for the sample and the reference measurements, respectively. IR-820 was used as the standard; Φ_F_ = 0.044 in methanol [25].

### 4.5. pH Studies

The effect of protonation on **2a** and **2b** was investigated through the addition of aliquots of a dilute TFA solution in CH_2_Cl_2_. 3000 µL of TFA in 7000 µL (6000 µL in the case of **2b**) of Millipore water was prepared and used for the pH studies. Concentrations of 3.2 × 10^−6^ and 3.6 × 10^−5^ M of **2a** and **2b**, respectively, were used in 4 mL of solution. A total of 50 µL of the TFA solution was added gradually, and changes in pH were monitored. The buffer solutions were prepared according to literature procedures [26] with slight modifications (Table S1, Supporting Information). A UV-visible absorption spectroscopy method was used to determine the pK_a_ value. Stock solutions of 1.5 × 10^−6^ and 1.9 × 10^−5^ M of **2a** and **2b**, respectively, were prepared in DMSO. The vials were filled with different buffers of constant ionic strength (*I* = 1 M), ranging from pH 0.4 to 9.0. A fixed amount of stock solution for both compounds was added to each vial and mixed, except in vials that were used as blank buffers to provide correction factors.

### 4.6. Theoretical Calculations

The Gaussian 09 software package [27] was used to carry out density functional theory (DFT) geometry optimizations at the B3LYP/6-31G(d) level of theory for **2a**, **2b** and a series of model complexes. Time dependent-DFT (TD-DFT) calculations were carried out by using the CAM-B3LYP functional with 6-31G(d) basis sets. The CAM-B3LYP functional was used since it contains a long-range correction that makes it more suitable for use with dyes that exhibit ICT character.

## 5. Conclusions

Two pH-sensitive NIR absorbing azaBODIPY dyes have been successfully synthesized and characterized. The introduction of electron-donating dimethylaniline and thien-2-yl groups results in unusually large red shifts of the main azaBODIPY absorption band well into the NIR region at 824 and 790 nm, respectively, in CH_2_Cl_2_. A larger red shift is observed for **2a**, since the more strongly electron-donating dimethylaniline rings lie at the 3,5-positions, which have large MO coefficients in the HOMO. A relative destabilization of the HOMO results in a narrowing of the HOMO–LUMO gap. In bulk solution, the dyes are only fluorescent under acidic conditions, due to suppression of the ICT process upon protonation. This provides scope for a pH-dependent fluorescence “turn-on” effect. **2a** with strongly electron-donating dimethylaniline rings at the 3,5-positions has a pK_a_ value of 6.9 (±0.05) and is more strongly acidic than **2b**, which has a pK_a_ value of 7.3 (±0.05). The monoprotonated species of **2a** and **2b** were estimated to have pK_a_ values of 1.4 (±0.05). The visible colorimetric changes under acidic conditions demonstrate that **2a** and **2b** are good candidates for use as pH indicators under strongly acidic conditions.

## Figures and Tables

**Table 1:** Wavelengths of the main spectral bands of **2a** and **2b** in a range of different solvents.

	DMSO	CH_2_Cl_2_	THF	Toluene	Benzene	Ref.
**1**	--- ^1^	---	---	798	---	[12]
**2a**	859	824	819	816	814	---
**2b**	810	790	786	776	779	---

^1^ No value available.

## Supplementary Materials

The following are available online. Figure S1: 1H NMR spectrum of 2a in acetone-d6, Figure S2: 1H NMR spectrum of 2b in CDCl_3_, Figure S3: MS data for 2a, Figure S4: MS data for 2b, Figure S5: Stacked 1H NMR spectra for 2b titrated with TFA in CDCl_3_, Table S1: Preparation of buffer solutions of constant ionic strength (*I* = 1 M), Table S2: Calculated electronic excitation spectra of **2a** and **2b** at the CAM-B3LYP/6-31G(d) level of theory.
