# Supplementary material for: NIR Absorbing AzaBODIPY Dyes for pH Sensing
Source: Molecules. 2020 Aug 13;25(16):3689. doi: 10.3390/molecules25163689 (PMC7465905; doi:10.3390/molecules25163689)
Supplement: Supplementary file 1 [file molecules-25-03689-s001.pdf]

## Supporting information

# NIR absorbing azaBODIPY dyes for pH sensing


**Gugu Kubheka [1], John Mack [1,*], Tebello Nyokong [1] and Zhen Shen [2,*]**

[1] Institute for Nanotechnology Innovation, Department of Chemistry, Rhodes University, Grahamstown 6140, South Africa; j.mack@ru.ac.za

[2] State Key Laboratory of Coordination Chemistry, School of Chemistry and Chemical Engineering, Nanjing University, Nanjing 210046, P. R. China; zshen@nju.edu.cn

* Correspondence: j.mack@ru.ac.za; Tel.: +27-46-603-7234 (J.M.): zshen@nju.edu.cn; Tel.: +86-25-8968-6679 (Z.S.)


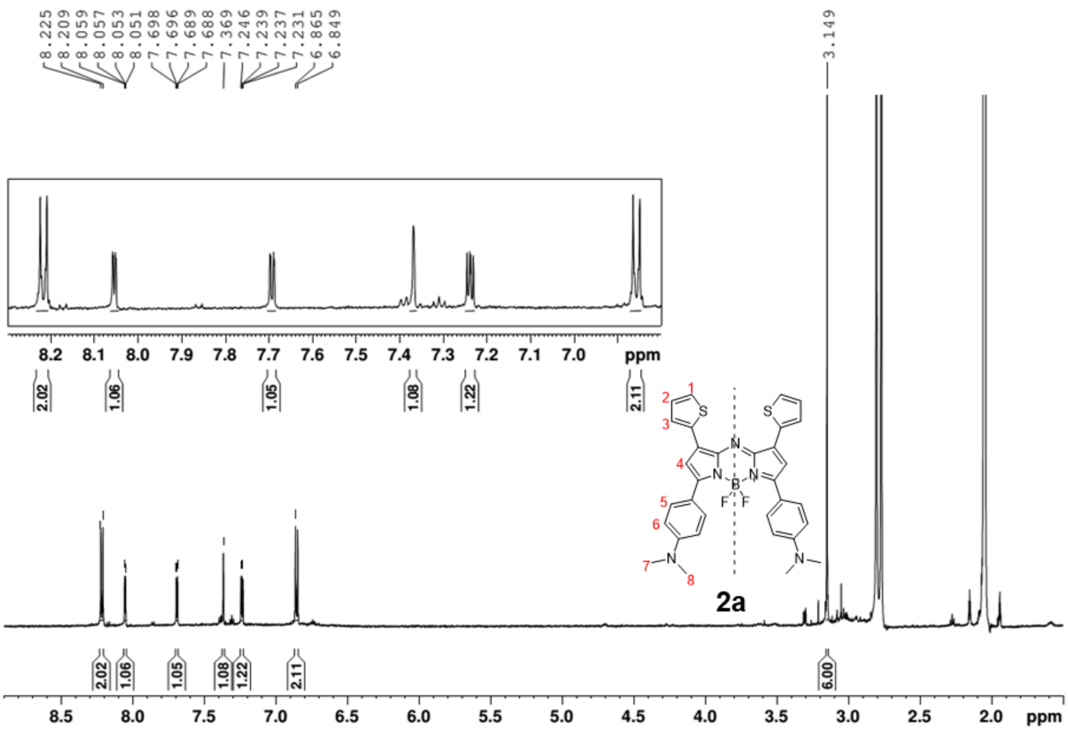


**Figure S1**: $^{1}$H NMR spectrum of **2a** in acetone-*d*6.

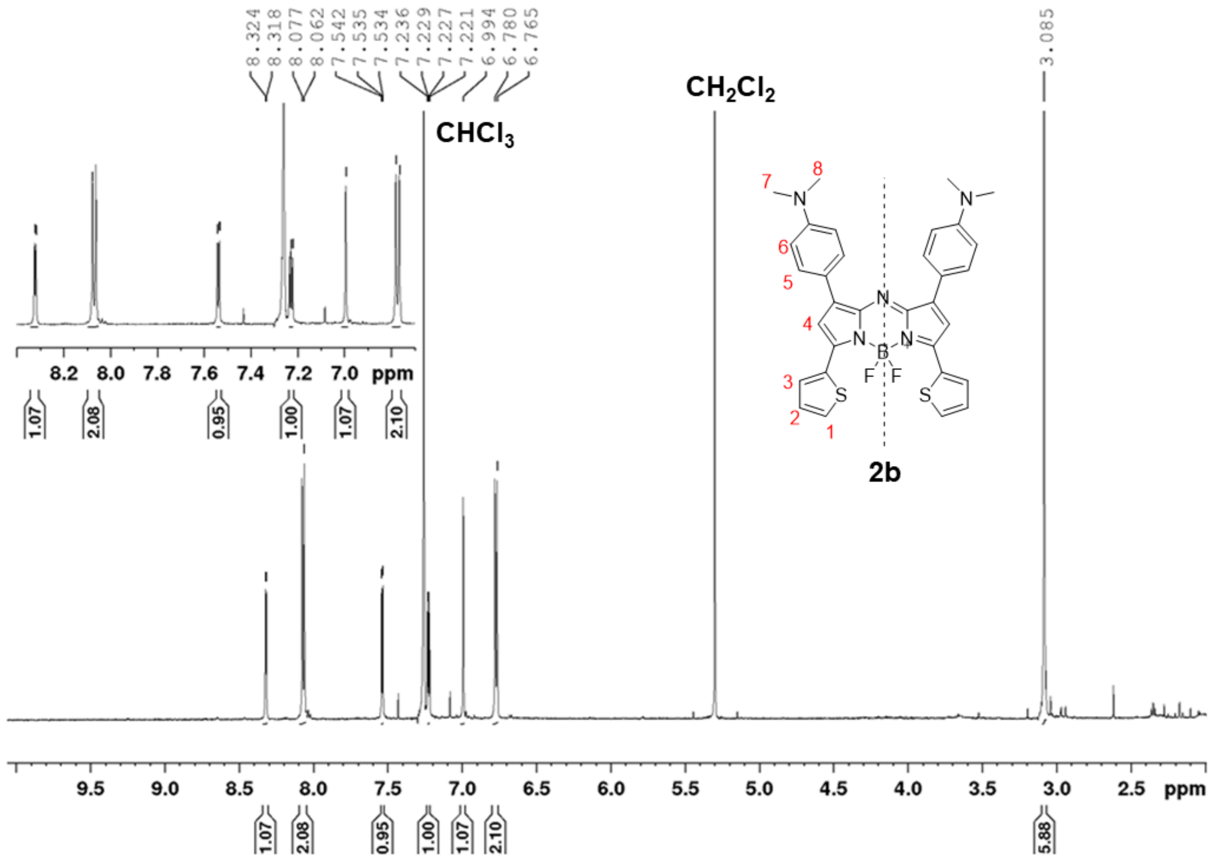


**Figure S2**: $^{1}$H NMR spectrum of **2b** in $CDCl_3$.

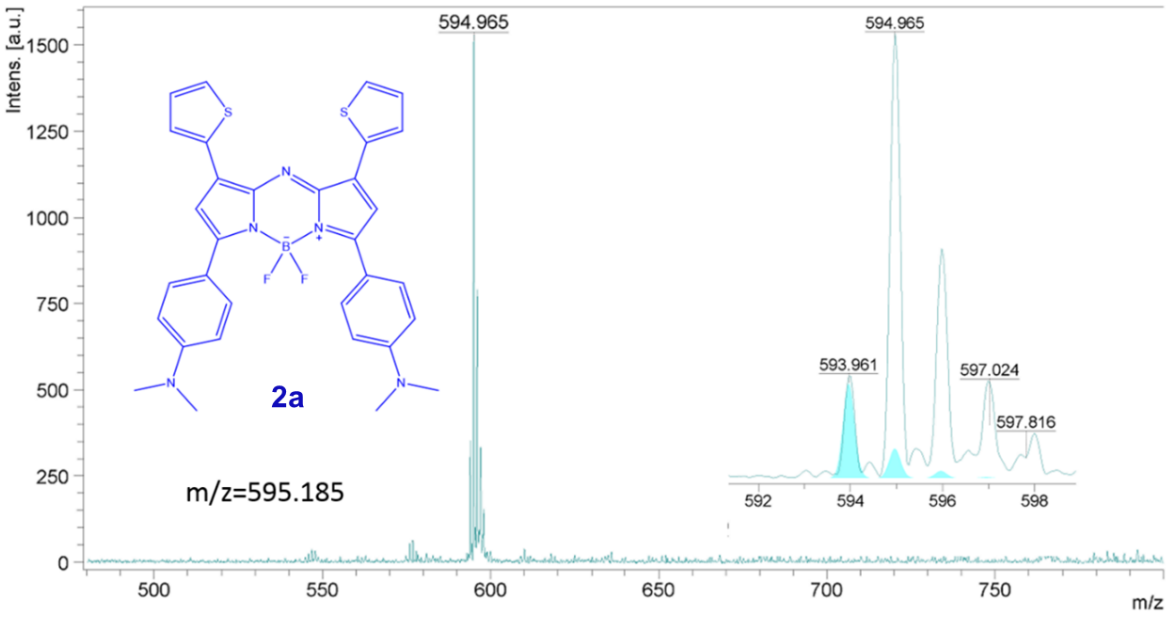


**Figure S3**: MS data for **2a**.

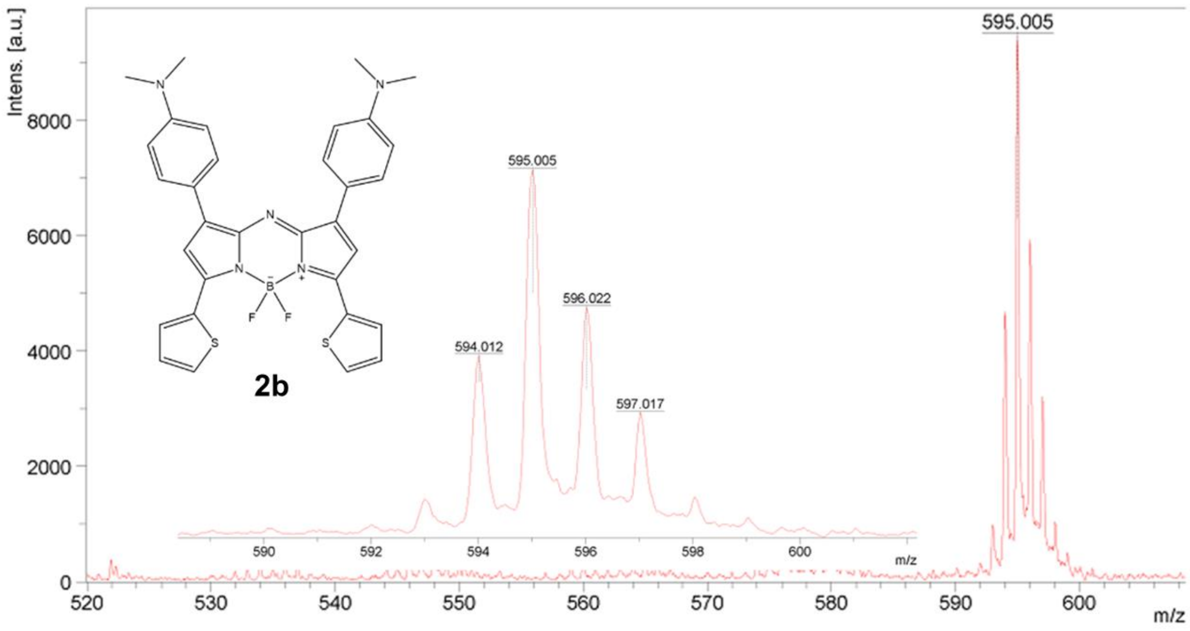


**Figure S4**: MS data for **2b**.

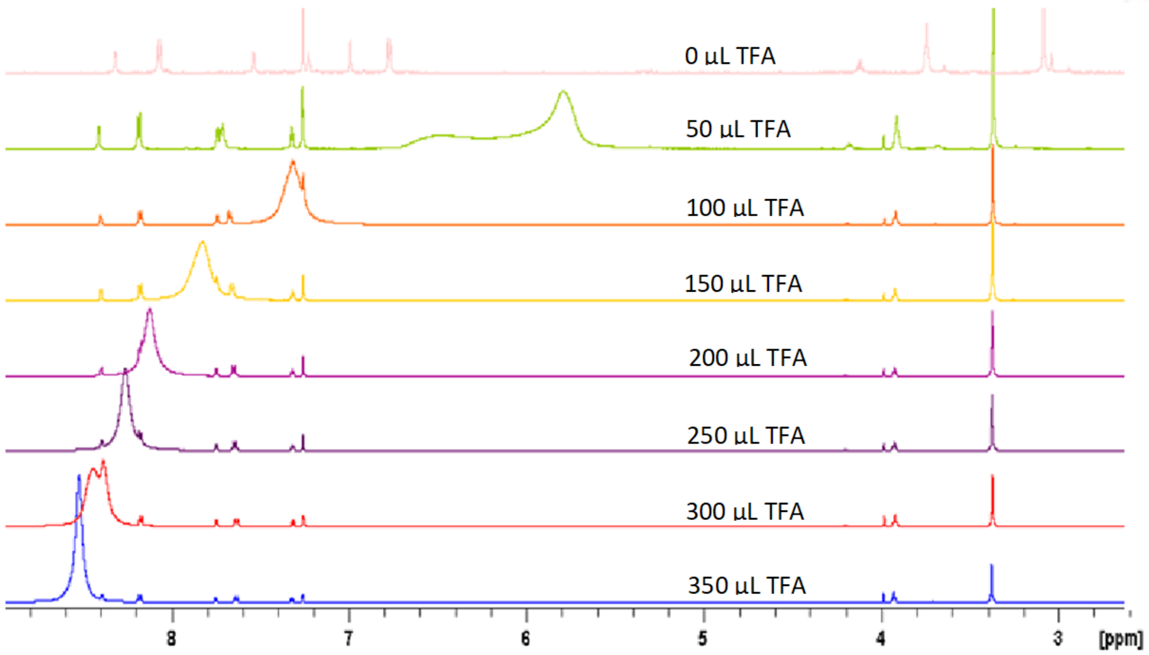


**Figure S5**: Stacked $^1$H NMR spectra for **2b** titrated with TFA in $CDCl_3$.

**Table S1**: Preparation of buffer solutions of constant ionic strength (*I* = 1 M). The pH values were measured at ≈ 20°C.

| *Buffer | Target pH | Measured pH | Conc. of Salt Solution (M) | Vol. of Salt Solution (ml) | Vol. of HCl (ml) | Vol. of NaOH (0.1 M) (ml) | Total Vol $H_2O$ (ml) | Calculated Ionic Strength (M) | Conc. of KCl required (M) |
|---|---|---|---|---|---|---|---|---|---|
| KCl/HCl | 0.40 | 0.40 | 2.0 | 25 | 48.6 | | 200 | 0.736 | 0.26 |
| | 0.60 | 0.62 | | 50 | 29.5 | | | 0.695 | 0.31 |
| | 0.80 | 0.82 | | | 11.5 | | | 0.515 | 0.49 |
| | 1.0 | 1.02 | | | 7.1 | | | 0.471 | 0.53 |
| KCl/HCl | 1.2 | 1.19 | 0.20 | | 3.6 | | | 0.436 | 0.56 |
| | 1.4 | 1.42 | | | 107.1 | | | 0.1571 | 0.84 |
| | 1.6 | 1.62 | | | 31.1 | | | 0.0811 | 0.92 |
| | 1.8 | 1.81 | | | 17.2 | | | 0.0672 | 0.93 |
| | 2.0 | 2.02 | | | 9 | | | 0.059 | 0.94 |
| $C_8H_5KO_4$ | 2.5 | 2.51 | | 100 | 69.9 | | | 0.2699 | 0.73 |
| NaOAC/ AcOH | 3.0 | 3.03 | 0.10 | 25 | 50.9 | | 100 | 0.0759 | 0.92 |
| | 4.0 | 4.03 | | | 38.3 | | | 0.0633 | 0.94 |
| | 5.0 | 5.08 | | | 13 | | | 0.038 | 0.96 |
| $KH_2PO_4$ | 6.0 | 6.00 | | | | 1.4 | | 0.0264 | 0.97 |
| | 7.0 | 7.00 | | | | 6 | | 0.031 | 0.97 |
| | 8.0 | 8.04 | | | | 11.2 | | 0.0362 | 0.96 |
| | 9.0 | 9.04 | | | | 19.05 | | 0.04405 | 0.96 |

*$C_8H_5KO_4$ (potassium hydrogen phthalate, NaOAC/AcOH (Sodium acetate/ acetic acid) and $KH_2PO_4$ (Potassium hydrogen phosphate)

**Table S2.** Calculated electronic excitation spectra of **2a** and **2b** at the CAM-B3LYP/6-31G(d) level of theory.

| #[a] | E[b] [eV] | $\lambda_{calc}$[c] [nm] | $f$[d] | $\lambda_{exp}$[e] [nm] | Wavefunction[f] |
|---|---|---|---|---|---|
| | | | | | **AzaBDY** |
| $S_1$ | 2.89 | 429 | 0.44 | --- | 92% HOMO → LUMO; ... |
| | | | | | **2a** |
| $S_1$ | 1.98 | 625 | 0.82 | 824 | 98% HOMO → LUMO; ... |
| $S_2$ | 3.08 | 402 | 0.80 | --- | 70% HOMO−1→ LUMO; 27% HOMO−3→ LUMO; ... |
| | | | | | **2b** |
| $S_1$ | 2.04 | 608 | 0.69 | 790 | 99% HOMO → LUMO; ... |
| $S_2$ | 2.69 | 461 | 0.43 | --- | 69% HOMO−1→ LUMO; 23% HOMO−2→ LUMO; ... |

[a]Excited state numbers in increasing energy from the TD-DFT calculations. [b]Calculated transition energies in eV. [c]Calculated wavelengths in nm. [d]Calculated oscillator strengths. [e]Experimental absorption maxima wavelengths in nm (solvent = DMSO). [f]Wavefunctions of the one-electron transitions involved in the transitions and their respective contributions on the basis of the eigenvectors predicted by TD-DFT calculations.
